# Long-term fertilization of a boreal Norway spruce forest increases the temperature sensitivity of soil organic carbon mineralization

**DOI:** 10.1002/ece3.895

**Published:** 2013-11-25

**Authors:** Elsa Coucheney, Monika Strömgren, Thomas Z Lerch, Anke M Herrmann

**Affiliations:** 1 Department of Chemistry, Uppsala BioCenter, Swedish University of Agricultural SciencesP.O. Box 7015, SE-75007, Uppsala, Sweden; 2 Department of Soil and Environment, Swedish University of Agricultural SciencesP.O. Box 7014, SE-75007, Uppsala, Sweden; 3 Biogéochimie et Écologie des Milieux Continentaux, UPECCréteil Cedex, 94010, France

**Keywords:** Boreal soils, microbial community, nutrient fertilization, soil organic carbon, temperature sensitivity, warming

## Abstract

Boreal ecosystems store one-third of global soil organic carbon (SOC) and are particularly sensitive to climate warming and higher nutrient inputs. Thus, a better description of how forest managements such as nutrient fertilization impact soil carbon (C) and its temperature sensitivity is needed to better predict feedbacks between C cycling and climate. The temperature sensitivity of in situ soil C respiration was investigated in a boreal forest, which has received long-term nutrient fertilization (22 years), and compared with the temperature sensitivity of C mineralization measured in the laboratory. We found that the fertilization treatment increased both the response of soil in situ CO_2_ effluxes to a warming treatment and the temperature sensitivity of C mineralization measured in the laboratory (Q_10_). These results suggested that soil C may be more sensitive to an increase in temperature in long-term fertilized in comparison with nutrient poor boreal ecosystems. Furthermore, the fertilization treatment modified the SOC content and the microbial community composition, but we found no direct relationship between either SOC or microbial changes and the temperature sensitivity of C mineralization. However, the relation between the soil C:N ratio and the fungal/bacterial ratio was changed in the combined warmed and fertilized treatment compared with the other treatments, which suggest that strong interaction mechanisms may occur between nutrient input and warming in boreal soils. Further research is needed to unravel into more details in how far soil organic matter and microbial community composition changes are responsible for the change in the temperature sensitivity of soil C under increasing mineral N inputs. Such research would help to take into account the effect of fertilization managements on soil C storage in C cycling numerical models.

## Introduction

Soils underpin the delivery of a wide range of ecosystem goods and services including climate and carbon regulation. Boreal ecosystems store about one-third of global soil organic carbon (SOC), which has been recognized as the most valuable of the ecosystem services supported by the boreal biome (Schindler and Lee 2010). Investigating the reaction of this major pool of organic C to climate and managements is crucial to be able to predict feedbacks between SOC and climate (Schmidt et al. 2011). This is especially important in boreal ecosystems because they are very sensitive to environmental changes such as higher nutrient deposition (Olsson et al. 2005; Allison et al. 2007) and climate warming (Bjerke et al. 2011; Contosta et al. 2011).

It was previously shown that considering C-nitrogen (N) interactions would strengthen our predictions about SOC-climate feedbacks in boreal ecosystems (Sokolov et al. 2008; Gärdenäs et al. 2010). For example, nutrient availability (including mineral N) and nutrient fertilization have been reported to affect boreal ecosystem response to warming (Rustad et al. 2001; Strömgren and Linder 2002; Xu et al. 2011). Moreover nutrient inputs to terrestrial ecosystems have dramatically increased since the beginning of the 20th century (Magnani et al. 2007; Eliasson and Ågren 2011), and the long-term effect on soil C remains unclear. These changes occur through man-induced environmental practices, that is, nutrient fertilization or increased N deposition (Vitousek et al. 1997; Janssens et al. 2010), and could also occur via the potential stimulation of N mineralization with climate warming (Rustad et al. 2001). Long-term nutrient fertilization experiments suggest that the effect of nutrient fertilization on ecosystem functioning is complex and may be seasonally dependent (Contosta et al. 2011). The addition of nutrients alters not only primary production (Hyvönen et al. 2007) but other ecosystem components such as plant and microbial diversity (Tilman 1987; Demoling et al. 2008), soil C storage and dynamics (Fog 1988; Olsson et al. 2005; Jandl et al. 2007; Phillips and Fahey 2007; Janssens et al. 2010) and N cycling (Lu et al. 2011). A recent meta-analysis showed that, in most cases, high N deposition in forest ecosystems reduces soil organic matter (SOM) decomposition, resulting in an increase in soil C sequestration in the long-term (Janssens et al. 2010). The reduction in SOM decomposition was often related to a decrease in the microbial biomass (Treseder 2008) or to some changes in the soil microbial community composition (Allison et al. 2007; Demoling et al. 2008; Campbell et al. 2010). Finally, it was recently reported that nutrient fertilization increased the temperature sensitivity of in situ soil C efflux (Q_10_ values) in wetlands (Jin et al. 2010) and the temperature sensitivity of C mineralization measured in laboratory conditions in tropical soils (Cusack et al. 2010). But the consequences of fertilization on C mineralization response to temperature have not been yet investigated in boreal soils. This is, however, of importance to evaluate whether such managements can modify SOC-climate interaction and should be taken into account in numerical models of SOC cycling (Schmidt et al. 2011).

In this study, we investigated the effect of long-term nutrient fertilization of a boreal ecosystem on temperature sensitivity of soil C cycling. We hypothesized that boreal forest fertilization modifies the temperature sensitivity of SOM decomposition due to the changes in SOM and/or in soil microbial communities that was previously observed (e.g., Swanston et al. 2004; Allison et al. 2007). Based on previous observations, we made the assumption that decomposition processes are slowed down in long-term fertilized soils (Janssens et al. 2010) possibly due to an increase in the stabilization of SOM (Swanston et al. 2004) and to some modifications in the soil microbial community (Allison et al. 2007). We further hypothesized that such changes may increase the sensitivity of C mineralization to temperature, as previously found in tropical soils (Cusack et al. 2010) and in turn to affect the overall ecosystem response to warming. To test these hypotheses, we investigated the sensitivity to warming of in situ soil C efflux measured during the growing season in a warming and long-term nutrient fertilization experiment of a boreal spruce forest in northern Sweden (Strömgren and Linder 2002) and the temperature sensitivity of soil C mineralization measured in the laboratory, as well as SOM characteristics and microbial community composition.

## Materials and Methods

### Experimental site

The Flakaliden warming experiment (Strömgren and Linder 2002) is located on a long-term nutrient fertilization experiment (Linder 1995) in northern Sweden (64°07′N; 19°27′E, 310 m). The site has a boreal climate with a mean annual temperature of 2.3°C; ranging from −8.7 in February to 14.4°C in July. About one-third of the annual precipitation (600 mm) falls as snow, which usually covers the ground from mid-October to mid-May. The forest stand consists of Norway spruce (*Picea abies* (L.) Karst) and was planted in 1963. The soil is a sandy loamy till and classified as a Haplic podzol (according to FAO, 1990) with an average depth of 120 cm.

The long-term nutrient fertilization experiment started in 1987 including *inter alia* a treatment with a complete nutrient solution (fertilized-irrigated, FI) and a control treatment with soil irrigation only (I). Fertilizer solutions were applied as a complete nutrient addition including micronutrients to maintain a nutrient target in the needles (see Linder 1995). The annual dose of N (NH_4_NO_3_) in fertilized treatment varied from 0 to 100 kg N/ha in the period 1987 to 2009, and in total, 1525 kg N/ha had been added by 2009. The other nutrients were supplied in fixed proportion to N. The control treatment received irrigation in order to avoid biases due to water stress. Both fertilizer solutions and water amendments maintained soil moisture potential above −100 kPa.

In 1995, a warming experiment was initiated on top of the fertilization experiment. Four 10 × 10 m plots were marked off from the main treatments (I, FI). Since then, two subplots were exposed to warming and were maintained at 5° (5 cm depth) above ambient temperature in their paired subplots during the vegetation period in May–October. Thus, the experimental field design consists of four treatments: control-irrigated (I), warmed-control-irrigated (WI), FI and warmed-fertilized-irrigated (WFI) with two field replicates. A large proportion of the soil profile was affected of the warming treatment. In 2009, from May to October, mean temperature differences of about 5° were observed from the organic layer to a depth of 40 cm in the mineral soil (Leppälammi-Kujansuu et al. 2013). Due to the irrigation treatment, the soil water content was not significantly different between control and warmed treatments over the growing period (Fröberg et al. 2013). For further details regarding the soil warming experiment, see Bergh and Linder (1999) and Strömgren and Linder (2002).

### In situ soil-surface CO_2_ flux and soil temperature

Throughout 2009, soil-surface CO_2_ fluxes (*R*) were measured in situ with an opaque chamber connected to a gas analyzer (SRC-1 with an EGM-4 and STP-1; PP systems, Hitchin, UK) approximately every third week during the growing season. Soil-surface CO_2_ flux included emissions from both autotrophic (ground vegetation) and heterotrophic respiration and was measured on three random locations for each of the replicated subplots. During each measurement, the respiration chamber was attached to preinstalled collars (diameter 10 cm) and the CO_2_ concentration was monitored every 4.2 sec over an 80-sec interval. After a phase of stabilization of the signal, the last 14 observations were used for estimating *R* through a linear fit. Soil temperature at 10 cm depth was measured adjacent to each measurement point (STP-1; PP systems). Measurements from a warmed subplot and its control plot were in general measured within an hour. The relationship between *R* and soil temperatures (10 cm depth) measured over the growing season was investigated by fitting data to a simple exponential model given by equation 1:


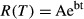
(1)

with *R* as the rate of soil-surface CO_2_ flux (*μ*mol/m^2^/sec), T as the temperature measured in the soil (°C), and A and b are constant parameters given by the fitting of the model. The temperature dependency of *R* was expressed as a seasonal Q_10_, which represents the proportional change in *R* given a 10°C change of temperature and that was derived from equation 1 and is given by equation 2:


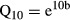
(2)

### Soil sampling and organic matter analysis

Soil samples were taken in September 2009 at the end of the vegetation period. Three soil cores were taken from the same locations where seasonal soil CO_2_ effluxes (see above) were recorded and collected on each of the two field replicates. Thus, in total six samples per treatment were sampled. Additionally, as soil chemical and biological processes differ with depth (Salome et al. 2010) and because the temperature sensitivity of SOM dynamics may also be different between soil horizons (von Lutzow and Kogel-Knabner 2009; Gillabel et al. 2010), the two upper soil horizons were sampled separately: (1) the organic O horizon corresponding to three subhorizons Oi, Oe, and Oa; and (2) the top 10 cm of the eluvial E horizon. Samples were sieved through a 4-mm sieve (i.e., material including fine roots that were smaller than 4 mm were kept) and stored frozen until further studies and analyses. C and N content of the samples were measured on dried samples by an elemental analyzer (LECO CN-2000).

### Microbial community composition analysis

The composition of the soil microbial community was assessed by phospholipid fatty acid analysis (PLFA) according to Frostegard et al. (1993). Briefly, lipids were extracted from approximately 0.5 and 2 g of the frozen O and E horizon, respectively, using chloroform, methanol, and citrate buffer to the ratio of 1:2:0.8 (v/v/v). Phospholipids were then fractionated by solid-phase extraction and subjected to mild alkaline methanolysis. The resultant fatty acid methyl esters (FAME) were detected and quantified with an Agilent/HP model 5890N gas chromatograph coupled to a flame ionization detector (GC-FID) using bacterial FAME standards (BAME Mix 47080-U; Supelco, Sigma Aldrich, St. Louis, MO). Methylnonadecanoic acid (Me 19:0) was used as internal standard. Further identification was obtained by analyzing the lipid fraction with an Agilent/HP model 5890N gas chromatograph coupled to a HP 5970 quadrupole mass spectrometer (GC-MS). Fatty acid nomenclature used was that described by Frostegard et al. (1993). Monounsaturated and cyclopropyl fatty acids were taken as gram-negative bacteria (G-) biomarkers (Zelles 1999), iso- and anteisofatty acids as gram-positive bacteria (G+) biomarkers (O'Leary and Wilkinson 1988), linoleic acid C18:2 (9,12) as a fungal biomarker (Frostegard et al. 1993) and carboxylic acids with a methyl function on the C chain as biomarkers for actinobacteria (Zelles 1997). Fungal-to-bacterial ratio (F:B ratio) was based on the abundance of the fungal PLFA biomarker 18:2 (9, 12) and the sum of 8 bacterial PLFA biomarkers (i-C15:0, a-C15:0, C15:0, i-C16:0, i-C17:0, a-C17:0; C17:0, and C19:0 d).

### Short-term incubation experiments and C mineralization

Approximately 60 and 140 g dry weight soil from the O and E horizons, respectively, were thawed for 5 h at 4°C. Soil samples were adjusted to 60% of their water-holding capacity and placed into 500-mL hermetically sealed jars with 10 mL water in the bottom of each jar to ensure humid conditions during the incubation. Samples were then preincubated for 3 weeks at constant temperature (15°C) to allow the microbial respiration flush from fresh organic matter released due to sampling and freezing procedure to subside (Herrmann and Witter 2002).

After the preincubation period, samples were split into four subsamples of 15 g for the O horizon and 40 g for the E horizon and incubated over a 10-day incubation period at four different temperatures (2, 5, 10, and 15°C, respectively). Evolved CO_2_ was trapped in a 0.5 mol/L NaOH solution and determined every second day by titration with 0.3 mol/L HCl after addition of BaCl_2_. Airtight jars without soil acted as controls. C mineralization rates were determined by a linear regression of CO_2_-C evolved over time during the whole incubation period. The temperature sensitivity of C mineralization data (Q_10_ values) was then calculated as described above (eq. 1 and 2).

### Statistical analysis

All statistical analyses were performed in R, version 2.9.1 (R Development Core Team 2008), using the “Vegan: Community Ecology Package” (Oksanen et al. 2011). The relative concentration of FAME (mol%) in the PLFA profiles was subjected to canonical correspondence analysis (CCA), which is a direct gradient analysis that maximizes the correlation between species (here, PLFAs) scores and sample scores while constraining sample scores to be linear combinations of explanatory variables (here, the fertilization and the warming treatments, respectively). The significance of the constraints was assessed using an analysis of variance (ANOVA)-like permutation test. Additionally, changes in microbial groups' abundance due to nutrient fertilization and warming were expressed relatively to the control-irrigated treatment (I), that is, ratio of abundances in the WI, FI, and combined FWI, respectively, to abundances in the I treatment were calculated.

Due to the experimental design, the differences in soil organic C and N, soil C:N ratio, soil CO_2_ efflux (*R*) and in microbial group abundances were tested within treatments (“main effects”) and between field replicates (“nested effects”) using a nested two-way ANOVA followed by Tukey's multiple pair test comparisons.

Because of high spatial variation in total SOC, C mineralization rates were normalized by the C content of the soil sample, that is, data were expressed per g C. Similarly, differences in soil C mineralization at different temperatures in the laboratory were tested within treatments (“main effects”), within temperatures (“main effects”), and between field replicates (“nested effects”) by a nested three-way ANOVA followed by Tukey's multiple pair test comparisons. We found no significant differences between field replicates except for the soil C content in the O horizon of the FI treatment. As a result, the values of the two field replicates were pooled together and represented as mean and standard error of six replicates in tables and figures, except for the C content in the O horizon of the FI treatment.

## Results

### In situ soil-surface CO_2_ fluxes during the growing season and temperature sensitivity of C mineralization

**Figure 1:**
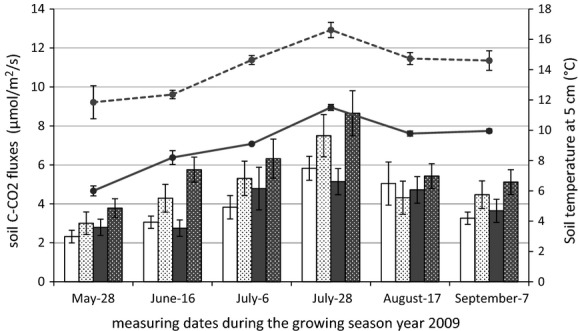
Soil-surface CO_2_ fluxes during the growing season in 2009 and before soil sampling (average and standard error; *n* = 3 spots × 2 blocks) from the control-irrigated (I, white bars), warmed-control-irrigated (WI, white-dotted), fertilized-irrigated (FI, gray bars) and combined warmed and fertilized-irrigated (WFI, gray-dotted) treatments and the respective soil temperatures measured at 10 cm depth for the control (full line) and warmed plots (dotted line), respectively.

The seasonal variation in soil-surface CO_2_ fluxes showed a classic pattern with lower fluxes in spring and autumn and higher fluxes at the peak of vegetation in July (Fig. 1). Both warming and fertilization increased the soil-surface CO_2_ fluxes during the growing season but to a higher extent for the fertilization treatment. For all measuring dates, the fluxes were largest in the combined WFI treatment compared with all other treatments (Fig. 1). The effect of the warming treatment was season dependent: It increased the soil-surface CO_2_ flux by 21%, 25%, and 12% in nonfertilized plots and by 26%, 19%, and 15% in fertilized plots in May, July, and September, respectively (Fig. 1). When fluxes were pooled over the season, the increase was only 2% for the nonfertilized plots, but 16% in the fertilized ones (Table 1). In addition, the seasonal Q_10_ values based on soil temperatures measurements were higher in the FI treatments compared with the control-irrigated treatments (I; Table 2).

**Table 1:** Total soil C respiration (i.e., soil CO_2_-C efflux in *μ*mol/m^2^/s) on the last date before sampling (7th September) or sum of the eight different measurements made over the growing season (i.e., SUM = sum of effluxes measured at each sampling dates: 28th May, 16th June, 6th July, 28th July, 17th August, 7th September, 30th September and 19th October), soil organic C and N (*μ*g/g soil), C:N ratio, and C mineralization rates measured during the 10-day incubation period at 15°C (*μ*g/g organic C) in the control-irrigated (I), warmed-control-irrigated (WI), fertilized-irrigated (FI), and combined warmed and fertilized-irrigated (WFI) treatments and for the two soil horizons, respectively (average and standard deviation; *n* = 2 field replicates × 3 subsamples = 6 samples; excepted for the C content in the O horizon of the FI treatment where there was significant difference between field replicates, then both field replicate values are presented and *n* = 3 samples)

	Soil field measurements		Soil laboratory measurements							
		
	CO_2_-C efflux (*R*)		O horizon				E horizon			
			
Treatments	7th September	SUM	C	N	C:N	CO_2_-C	C	N	C:N	CO_2_-C
I	3.3 (0.3)^a^	23.3 (2.9)^a^	15.9 (2.1)^a^	0.50 (0.06)^a^	31.9 (2.1)^a^	658 (97)^a^	2.7 (0.5)^a^	0.10 (0.02)^a^	27.7 (0.8)^a^	689 (125)^a^
WI	3.6 (0.6)^a^	23.8 (3.5)^a^	16.9 (4.3)^a^	0.55 (0.12)^a^	29.4 (2.3)^a^	530 (95)^a^	2.3 (0.1)^a^	0.10 (0.01)^a^	23.7 (1.0)^a^	745 (75)^a^
FI	4.5 (0.7)^a^	28.9 (4.1)^a^	45.3/37.1 (3.3–1.7)^b^	1.47 (0.11)^b^	28.6 (0.4)^a^	587 (102)^a^	3.9 (0.5)^b^	0.15 (0.02)^b^	25.9 (0.6)^a^	653 (62)^a^
WFI	5.1 (0.6)^a^	33.6 (4.1)^a^	33.0 (3.5)^b^	1.16 (0.11)^b^	28.4 (1.2)^a^	636 (110)^a^	3.1 (0.6)^b^	0.12 (0.03)^b^	26.1 (1.8)^a^	764 (75)^a^

Different letters show different groups of mean values by column.

**Table 2:** Comparison between the seasonal temperature relationship of in situ measurements for soil C emissions and of the temperature sensitivity of C mineralization data of spatially paired samples expressed as Q_10_ values for the control-irrigated treatment (I) and the fertilized-irrigated treatment (FI), respectively (mean and standard error in brackets of *n* = 2 field replicates × 3 subsamples = 6 samples, because there were no significant differences between field replicates). Field temperature measurements were made at 10 cm depth

		Q_10_ for C mineralization	
		
	Seasonal Q_10_ for in situ C-CO_2_ fluxes	O horizon	E horizon
I	4.1 (0.5)	2.8 (0.2)	2.8 (0.2)
FI	4.8 (0.6)	3.4 (0.1)	3.6 (0.3)

**Figure 2:**
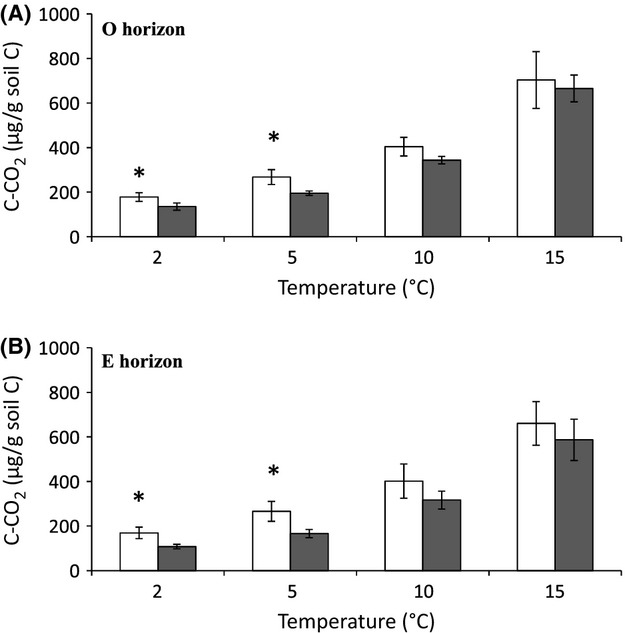
C mineralization rates estimated under laboratory conditions at 4 temperatures (2, 5, 10 and 15°C) for (A) the O horizon and (B) the E horizon from the control-irrigated (I, white bars) and fertilized-irrigated (FI, dark gray) treatments, respectively. *denotes significant differences between the two treatments (*P* < 0.05).

C mineralization rates obtained from the incubation study and measured at 15°C were not significantly different between the two soil horizons and within treatments (Table 1 and Fig. 2). They increased with increasing temperature similarly in the two soil horizons (Fig. 2). C mineralization rates were significantly lower in the FI soils compared with control-irrigated soils (I) when soils were incubated at low temperatures, that is, 2 and 5°C. This resulted in a significantly (*P* < 0.05) higher temperature sensitivity of C mineralization rates in the FI soils (Q_10_ = 3.4 and 3.6 for O and E horizons, respectively) compared with I soils (Q_10_ = 2.8 for both soil horizons; Table 2). Moreover, we found that the relative increase in temperature sensitivity of C fluxes (Q_10_) in FI treatments compared with control-irrigated treatments (I) was in the same order of magnitude for Q10 values obtained in the field and in the laboratory (Table 2).

### Soil organic matter

Soil organic matter content in the O horizon was 5–10 times higher than in the E horizon, and the C:N ratio was also significantly higher in the O horizon (*P* < 0.05; Table 1). Fertilization increased the C and N content of the two soil horizons, but did not change the C:N ratio of the SOM. On the other hand, the warming treatment did not change significantly SOM content and the C:N ratio. Nevertheless, the C:N ratio of SOM tended to be less in the warmed soils compared with the nonwarmed soils in both O and E horizons of the control-irrigated treatments (I and WI, Table 1). Soil pH was not significantly affected by the treatments and had an average value of 4.2. Only for the soil C content, there were significant differences between replicated treatments in the field (*P* < 0.05).

We found that soil-surface CO_2_ fluxes measured in situ were slightly but significantly correlated with the C content of the O and E horizon, respectively, *r*^2^ = 0.47 and 0.35 (*P* < 0.05; results not shown), but not to the C mineralization rates measured in the laboratory expressed per g of soil C. When C mineralization rates were expressed per g of soil, fertilization increased the mineralization rates by 118% and 19% in the O and E horizons, respectively, while it increased the soil-surface CO_2_ flux by 31% only (Table 1).

### Soil microbial community

**Figure 3:**
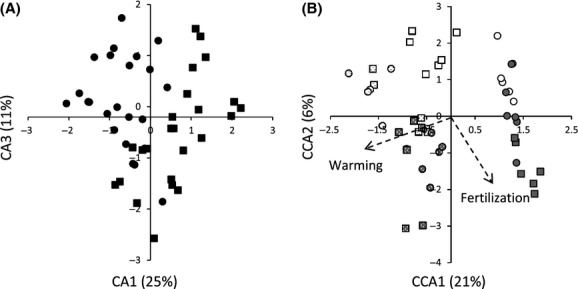
Differences in the phospholipid fatty acid analysis profiles of (A) the O (circles) and E (squares) soil horizons and (B) for the different treatments shown by soil samples scores along different canonical axes of the constrained canonical analysis (CCA). (A) O and E horizons were discriminated along the first and third nonconstrained axes, while (B) significant effect of the fertilization and warming treatments (*P* < 0.05) were revealed along the two constrained axes by discrimination of sample scores from the control-irrigated soils (I, white), warmed-control-irrigated (WI, white-dotted), fertilized-irrigated (FI, gray), and combined warmed and fertilized-irrigated (WFI, gray-dotted) treatments, respectively.

Differences between PLFA profiles of the two soil horizons were visible along the nonconstrained canonical (CA) axes 1 and 3 that explained 36% of total variability (Fig. 3A). In both soil horizons, soil microbial communities were significantly affected by the fertilization and the warming treatments as shown by the separation of samples scores along the two constrained (CCA) axes of the CCA that explained 27% of total variability (Fig. 3B).

**Figure 4:**
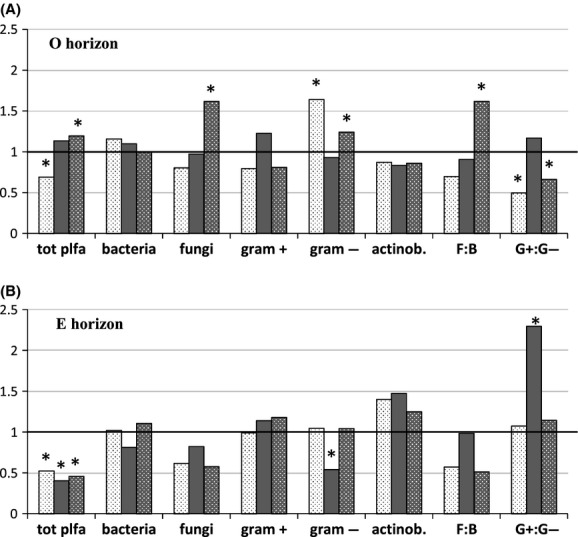
Ratios and abundances of main microbial groups in the warmed-control-irrigated (WI, white-dotted), fertilized-irrigated (FI, gray), and combined warmed and fertilized-irrigated (WFI, gray-dotted) treatments relative to the control-irrigated treatment (I; black line) for (A) the O horizon and (B) the E (below) horizon, respectively. *indicates significant differences (*P* < 0.05) between the control and one of the other treatments, respectively.

The effects of the fertilization and of the warming treatments on the relative abundances of different microbial groups were different for the two soil horizons (Fig. 4). In the O horizon, the combined WFI treatment had the strongest effect on the community composition: It increased the total microbial biomass, the fungal biomass, and the gram-negative bacterial biomass as well as it increased the ratio of fungal-to-bacterial biomass (Fig. 4A). The FI treatment had no significant effect on the relative abundances of microbial groups. The WI treatment increased the gram-negative bacterial biomarker, but decreased the total biomass and the ratio of gram-positive to gram-negative bacteria. In the E horizon, the FI treatment affected most of the microbial groups by decreasing the total microbial biomass and the gram-negative bacteria as well as increasing the ratio of gram-positive to gram-negative bacteria (Fig. 4B). The warming treatment resulted in less microbial biomass, irrespectively of fertilization treatments.

**Figure 5:**
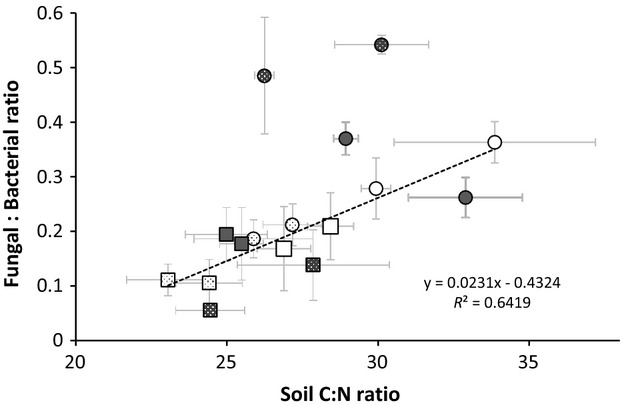
Relationship between the fungal-to-bacterial biomass ratio derived from phospholipid fatty acid analysis biomarkers and the soil C:N ratio (average and standard error, *n* = 3 soil samples) for the O (circle) and E (square) soil horizons from the two replicated plots of the control-irrigated (I, white), warmed-control-irrigated (WI, white-dotted), fertilized-irrigated (FI, gray), and combined warmed and fertilized-irrigated (WFI, gray-dotted) treatments, respectively. The dashed line represents the linear regression between the two variables when samples from the O horizon of the WFI treatment were excluded from the analysis (*P* < 0.05).

A significant relationship was found between the soil C:N ratio and the fungal-to-bacterial biomass ratio between the different soil horizons and the different soil treatments when samples from the O horizon of the FWI treatment were excluded (Fig. 5). This relationship was the result of (i) a higher C:N ratio and a higher fungal/bacterial biomass ratio in the O compared with the E horizon; and (2) a lower C:N ratio and a lower fungal/bacterial biomass ratio in the WI and WFI soils compared with the I and FI ones.

## Discussion

Long-term fertilization management of a boreal forest increased the temperature sensitivity of soil C mineralization (Figs. 1 and 2, Tables 1 and 2) as previously observed in tropical soils (Cusack et al. 2010). We hypothesized that such an increase in temperature sensitivity may be due to (1) changes in SOM and/or (2) alteration of microbial community composition when amending soil systems with long-term fertilization.

### Soil organic matter

We found that the soil C was increased in the fertilized treatments (Table 2), which suggests a change in the quantity and possibly in the stabilization of the SOM. These results are in line with a meta-analysis on the effects of increased N deposition on forest soil C reporting that soil C stocks were increased and heterotrophic respiration rates were reduced in 37 of 40 studies (Janssens et al. 2010). Changes in SOM quantity or stability have a strong potential to influence the temperature sensitivity of its decomposition (Agren and Bosatta 2002). For example, the decomposition of older organic matter has been shown to be more temperature sensitive than the labile organic matter (Biasi et al. 2005). In the study by Cusack et al. (2010), fertilization reduced oxidative enzymes that in turn decreased the decomposition of the more stable pool of SOM. Studies held on temperate forests also found an increase in SOM stabilization (Swanston et al. 2004) as well as an accumulation of the older organic matter following long-term N addition (Magill and Aber 1998; Franklin et al. 2003). On the other hand, nuclear magnetic resonance (NMR) data showed no major changes in C and N structure of the litter and SOM consecutive to long-term N fertilization of a boreal forest (Sjöberg et al. 2004). In the present study, we found no relationship between C:N ratio, C mineralization, and the temperature sensitivity of C mineralization. However, differences in SOM stability between long-term fertilized and control soils are most likely to exist according to previous studies (Magill and Aber 1998; Franklin et al. 2003; Swanston et al. 2004) but may involve SOM minerals' interactions rather than chemical recalcitrance (Moni et al. 2010).

### Microbial community composition

The temperature sensitivity of SOM mineralization is dependent on enzymatic activities and production by soil microbial communities (Cusack et al. 2010; Conant et al. 2011). In the boreal soil investigated here, the microbial community composition was significantly affected by both fertilization and warming (Fig. 3B). This is in line with previous studies held on boreal (Allison et al. 2007; Högberg et al. 2007; Demoling et al. 2008; Frey et al. 2008; Campbell et al. 2010; Long et al. 2012) or subarctic ecosystems (Rinnan et al. 2007). Furthermore, microbial community composition in the O and E horizons was different (Fig. 4), and thus, the effect of fertilization and warming was horizon specific, corroborating findings of community compositions along other podzol profiles (Fritze et al. 2000). Such differences may be related to C content and soil texture in the two horizons (Table 2). In the E horizon, fertilization decreased (1) the amount of total biomass as reported in previously published meta-analyses (Högberg et al. 2007; Demoling et al. 2008; Treseder 2008) and (2) the gram-negative bacteria relative abundance (Fig. 4B). In the rhizosphere, gram-negative bacteria are more common than gram-positive bacteria (Demoling et al. 2008), and their relative decrease in fertilized soils has been hypothesized to relate to a decrease in C allocation via roots with increasing N loading (Cannell and Dewar 1994).

In contrast with previous studies (Frey et al. 2004; Demoling et al. 2008), fertilization alone had no effect on the fungal-to-bacterial biomass ratio. In those studies, N fertilization was found to have a negative effect on ectomycorrhizal fungi because they constitute a group of soil microbes that are sensitive to the production of photosynthates that should be reduced under N addition (Högberg et al. 2003; Demoling et al. 2008). However, a meta-analysis (Treseder 2004) showed that the effects of N deposition on mycorrhiza communities are difficult to predict for individual ecosystems as the variation is quite high within studies. Moreover, in a recent study held on the same boreal forest as our study, the ectomycorrhizal root tips were found to be most abundant in FI versus control-irrigated soils (Leppälammi-Kujansuu et al. 2013).

The warming treatment in the field also changed the microbial community composition confirming findings proposed by Frey et al. (2008). It increased the relative abundance of gram-negative bacteria, but decreased total microbial biomass (Fig. 4B). The decrease in microbial biomass may be due to the depletion of easily decomposable SOM, which has been shown in other experimental warming studies (e.g., Hartley et al. 2007; Bradford et al. 2008). Interestingly, the modifications of the microbial community composition in the combined warmed and FI treatment were specific and were not the sum of independent effects from fertilization and warming (Figs. 3 and 4). This was also the case for oxidizing bacterial and archaeal communities investigated in the same ecosystem (Long et al. 2012). The fungal biomass and the fungal-to-bacterial biomass ratio in the O horizon were increased in the combined warmed and FI treatment only (Fig. 4A). This result may be linked to higher ectomycorrhizal root tips found in this treatment (Leppälammi-Kujansuu et al. 2013) but can also be due to an increase in the biomass of fungal decomposers.

Interestingly, we found that the differences in soil C:N ratio within samples were correlated with the fungal-to-bacterial biomass ratio when samples from the combined warmed-fertilizer-irrigated treatment were removed (Fig. 5). The soil C:N ratio is known to affect microbial composition in boreal soils (Högberg et al. 2007), and the correlation suggests that fertilization or warming only did not change significantly the relationship between soil C:N ratio and fungal-to-bacterial biomass ratio while the combination of warming and fertilization did. Those results revealed that the interactions between higher N input and soil warming have significant effect on the relationship between soil biogeochemistry and microbial communities and it is not only the sum of several effects.

Even if both the microbial community composition and the temperature sensitivity of C mineralization varied between soil treatments, we found no direct relationship between the two. More specifically, despite that the soil microbial community composition was different between soil treatments but also among soil horizons, we found consistent results on the effect of long-term N fertilization, which reduced SOM decomposition and increased its sensitivity to temperature. Similarly, soils along a latitudinal gradient had different microbial communities but the same temperature sensitivity of C turnover in a boreal ecosystem (Vanhala et al. 2008). Together, these results suggest that there is no direct relationship between microbial community composition and the temperature sensitivity of C mineralization in such ecosystems.

### Temperature sensitivity of in situ soil respiration versus laboratory C mineralization

Not many field studies have been investigating the combined effects of warming and nutrient fertilization on soil respiration and biology (e.g., Strömgren 2001; Contosta et al. 2011; Leppälammi-Kujansuu et al. 2013). At Flakaliden 6 years after the start of the experiment, Strömgren (2001) found that soil warming increased total soil respiration but there was no effect of fertilization. Similarly, after 2 years of experiment, soil respiration was increased in warmed treatments and to a lower extent in fertilized treatments in a hardwood forest, but again neither additive nor interactive effects of the two treatments were revealed (Contosta et al. 2011). Here, after 16 years of soil warming at the Flakaliden experiment, we found slightly different results: Warming still increased total soil respiration but to a lower extent than fertilization only. This increase in soil respiration in fertilized plots is probably due to (1) both an increase in underground plant respiration, as the fine root biomass was higher in these treatments (Leppälammi-Kujansuu et al. 2013), and (2) an increase in heterotrophic respiration as previously found by Vallack et al. (2012).

Moreover, an interaction between the two treatments was found: The warming treatment increased soil respiration in the FI soils more than in the control-irrigated ones (Fig. 1), suggesting an increase in temperature sensitivity of soil respiration in fertilized soils. Indeed, estimated Q_10_ values were similar between in situ measurements in the field and C mineralization rates obtained in the laboratory. The former includes both autotrophic (roots and ectomycorrhizal respiration) and heterotrophic respiration, whereas the latter is solely based on heterotrophic microbial activities. Previously, it has been suggested that N fertilization and temperature affect the autotrophic and heterotrophic part of total soil respiration differently. Vallack et al. (2012) discovered that N fertilization decreased ectomycorrhizal respiration in relation to root and heterotrophic respiration. Furthermore, it has been suggested that there is a lack of temperature response of mycorrhizal mycelium as well as root respiration (Moyano et al. 2008; Heinemeyer et al. 2007, 2011). Although our experimental design was not based on evaluating the different parts in soil respiration, similarity in Q_10_ values obtained under field and laboratory conditions suggest that either (1) heterotrophic and autotrophic respiration are similarly affected by changes in temperature or (2) heterotrophic respiration dominates total respiration. Yet, the data presented are not sufficient to conclude how much all respiration parts were affected by temperature. However, the heterotrophic part of respiration has often been observed as a substantial contribution to total respiration ranging from 60% to 65% (Moyano et al. 2008; Schindlbacher et al. 2009; Heinemeyer et al. 2011). We could thus hypothesize that the increase in the temperature sensitivity of C mineralization obtained specifically in the laboratory may be partly responsible for a higher sensitivity to warming of in situ total soil respiration in the FI treatment, especially at the end of the growing season when autotrophic respiration is declined (Heinemeyer et al. 2007).

## Conclusion

Our study confirmed that C dynamics may be more sensitive to an increase in temperature in boreal soils under long-term fertilization management in comparison with nutrient poor boreal soils. Those results could have significant effect on soil C storage in the long term under a warmer climate and suggest that the effects of forest managements such as nutrient fertilization on C cycling should be taken into account in soil C cycling numerical models. But, the causes of this increase are uncertain as there is no clear link between changes in SOM, microbial community composition, and temperature sensitivity. Different mechanisms and their combinations may be held responsible for such a result. They include combined changes in SOM stability and microbial community composition that in turn may affect different processes that are sensitive to temperature: that is, enzymatic depolymerization, substrate availability, and interactions between SOM and soil minerals (Conant et al. 2011). Can the change in the relation between SOM (C:N ratio) and microbial composition (fungal-to-bacterial ratio) found here explain the shift in the temperature sensitivity of C mineralization between control and fertilized soils? Further research is needed to investigate how far those different mechanisms are responsible for the change in the temperature sensitivity of SOM decomposition under increasing mineral N inputs.
